# Genetic Mapping of Quantitative Trait Loci Underlying Flowering Time in Chrysanthemum (*Chrysanthemum morifolium*)

**DOI:** 10.1371/journal.pone.0083023

**Published:** 2013-12-11

**Authors:** Fei Zhang, Sumei Chen, Jiafu Jiang, Zhiyong Guan, Weimin Fang, Fadi Chen

**Affiliations:** 1 College of Horticulture, Nanjing Agricultural University, Nanjing, China; 2 Jiangsu Province Engineering Laboratory for Modern Facility Agriculture Technology & Equipment, Nanjing, China; Nanjing Forestry University, China

## Abstract

Flowering time is an important trait in chrysanthemum, but its genetic basis remains poorly understood. An intra-specific mapping population bred from the cross between the autumn-flowering cultivar ‘Yuhualuoying’ and the summer-flowering ‘Aoyunhanxiao’ was used to determine the number and relative effect of QTL segregating for five measures of flowering time. From flowering time data recorded over two consecutive seasons, 35 additive QTL were detected, each explaining between 5.8% and 22.7% of the overall phenotypic variance. Of these, 13 were detected in both years. Nine genomic regions harboring QTL for at least two of the five traits were identified. Ten pairs of loci epistatically determined the flowering time, but their contribution to the overall phenotypic variance was less than for the additive QTL. The results suggest that flowering time in chrysanthemum is principally governed by main effect QTL but that epistasis also contributes to the genetic architecture of the trait, and the major QTL identified herein are useful in our ongoing efforts to streamline the improvement of chrysanthemum via the use of molecular methodology.

## Introduction

A number of environmental factors are recognized as important for the regulation of flowering time in plants. Some species have a particular requirement for a given photoperiod [1] and/or a sufficient period of exposure to low temperature [2] before the switch from vegetative to reproductive growth is triggered. More generally, ambient temperature [3,4] and nutrient status [5] have a major influence over the timing of flowering, acting via the modulation of phytohormone status [6]. In addition to the major effects of photoperiod and/or vernalization responsiveness, intraspecific genetic variation in flowering time is typically under polygenic control [7-11]. 

Chrysanthemum (*Chrysanthemum morifolium*) is a leading ornamental species, used as source of cut flowers, as pot plants, and for gardening and landscaping; its medicinal usage has also been documented [12,13]. Breeding activity has produced as many as 6,000 cultivars thus far [14]. Given that the plant's major feature is its inflorescence, its flowering time is an important trait of breeding interest, and is often of vital significance to its future landscaping and marketing possibilities. The flowering of chrysanthemum is generally sensitive to photoperiod and/or to ambient temperature; most cultivars flower in autumn under short-day induction, some others flowering summer are controlled by temperature [15,16]. The molecular mechanisms involved in flowering of chrysanthemum have received great attention in past years. An *FLORICAULA/LEAFY* homologous gene *CmFL* was reported to play an important role in gibberellins’ mediating flowering time of chrysanthemum [17], and the over-expression of *AP1*-*like* genes under short-day conditions induces early flowering in transgenic chrysanthemum plants [18]. Recently, *FLOWERING LOCUS T*-*like* genes (i.e. *FTL3*) was found to be a key regulator of photoperiodic flowering in chrysanthemum [19] and also its signaling reduction from the leaves to the shoot tip at high temperatures results in flowering retardation [20]. The latest research shows that an antiflorigen (*CsAFT*) produced in the leaves under a non-inductive photoperiod systemically inhibits flowering in chrysanthemum, thus preventing precocious flowering and enabling the year-round supply of marketable flowers by manipulation of day length [21]. In addition, an *NRRa* orthologous gene (viz. *CmNRRa*) involved in nutrition response and root growth acts to negatively regulate flowering time in chrysanthemum [14]. All these findings suggested that flowering of chrysanthemum is a complex trait under the control of multi genes. 

Despite the commercial importance of this trait, little was known of its mode of inheritance [22,23], even though it has been known for many years that the trait has a high level of broad sense heritability [24]. Recent results from mixed inheritance model of major gene plus polygene suggest that some measures of flowering time are governed by major genes [25,26], and a quantitative trait locus (QTL) analysis carried out in a population bred from the cross ‘Yuhualuoying’ x ‘Aoyunhanxiao’ succeeded in detecting the presence of a small number of genomic regions harboring genes affecting the duration of flowering [27]. These findings suggest that flowering time of chrysanthemum is quantitatively inherited but still far from being well understood. Among ornamental species, the most extensive application of QTL analysis to determine the genetic control of flowering time has been carried out in rose [28-30], providing a good example of dissecting inheritance pattern of flowering time in ornamental plants of high heterozygosity, polyploidy and asexual propagation. 

In chrysanthemum, flowering time is often characterized as squaring, bud coloring, initial flowering, full flowering and wilting. The present study was undertaken to define how much genetic variation exists in the flowering time traits in an inter-specific hybrid population of chrysanthemum, to identify the location and size of the QTL affecting flowering time segregating in a cross between an autumn- and a summer-flowering cultivar, and to clarify the correlations which obtain between a set of distinct flowering time traits. This study adds more knowledge on the genetic pattern of flowering time in chrysanthemum. 

## Materials and Methods

### Plant materials

The mapping population comprised 142 F_1_ progeny from the cross between chrysanthemums ‘Yuhualuoying’ and ‘Aoyunhanxiao’ by artificial hybridization in 2006. The former cultivar flowers in the autumn, while the latter flowers during the summer. Both cultivars (2n=6*x*=54) were bred by the Chrysanthemum Germplasm Resource Preserving Centre, Nanjing Agricultural University, China. For the different flowering time, the pollens were collected from the male parent ‘Aoyunhanxiao’ in summer, and then stored at -20°C in preparation for manual hybridization with the female parent ‘Yuhualuoying’ in autumn. Capitula of the female parent were emasculated by removing the inner hermaphroditic disk florets, and the ray florets were docked to expose the stigma. The emasculated capitula were covered in a paper bag for two days, after which the stored pollens of the male parent were transferred to the female parent using a brush. Pollinated inflorescences were re-enclosed in a paper bag to prevent uncontrolled pollination. Owing to the self-compatibility of female parent, the F1 progenies should undoubtedly be true hybrids. 

### Field trial and trait investigation

The parental cultivars and the mapping population were grown in the field at Nanjing during 2008 and 2009. Plants were propagated by cutting in April of each year, and a month later rooted cuttings were transplanted to the field in three fully randomized block replications, with each genotype repeated 6 times per replication. The block size was 1 m x 60 m, and the distance between adjacent plants was 35 cm. The plants were managed following standard commercial practice. Observations were recorded for the number of days to squaring (DS), to coloring (DC), to initial flowering (DIF), to full flowering (DFF) and to wilting (DW) from transplanting. Squaring was taken as the day when approximately 50% of shoots were in bud; coloring as the day when approximately 50% of the buds became pigmented; DIF as the number of days between transplanting and when approximately 50% of the buds were half open and fully pigmented; DFF as the number of days between transplanting and when approximately 50% of the buds were fully open; and DW as the number of days between transplanting and when approximately 50% of the flowers had wilted. A total of 9 randomly selected plants per genotype were measured for the five flowering time traits, respectively in 2008 and 2009, and the average values were used in the statistical analysis and QTL mapping. Statistical analyses of these data, including a Pearson correlation analysis, were carried out using the software package SPSS v13.0 (Chigaco, USA). The broad sense heritability for each trait was calculated following Knapp et al. [31]. The means of the two-year phenotypic data have been used for genetic analysis via mixed inheritance model of major gene plus polygene in another research [27]. 

### QTL mapping

The genetic maps of chrysanthemum cultivars ‘Yuhualuoying’ and ‘Aoyunhanxiao’ were previously constructed by Zhang et al. [32], using double pseudo-testcross mapping strategy. The maps were mainly composed of 675 sequence related amplified polymorphism (SRAP) markers, covering >1,900 cM with a mean inter-marker distance of < 7.0 cM. In this paper, only the linkage groups associated with the QTL segregating for flowering time traits of chrysanthemum were presented. 

Two separate QTL mapping analyses were performed using WinQTLCartographer v2.5 [33], applying the composite interval mapping (CIM) procedure [34]. The window size was set at 10 cM and the walking speed at 1 cM. The LOD threshold applied was 2.5. The contribution ratio by each additive QTL was calculated as the percentage of variance explained by each QTL in proportion to the total phenotypic variance, which could be obtained from the CIM results. The program QTLNetwork v2.0 [35] was subsequently used to identify epistatic QTL, based on combined phenotypic data of combined two years, and applying a mixed-model based composite interval mapping method [34] with a window size of 10 cM and a walking speed of 1 cM. A 10 cM filtration window was used to distinguish whether or not a pair of adjacent test statistic peaks indicated the presence of two distinct QTL. A set of 1,000 permutations was applied to each trait to calculate a critical F value at P < 0.05, and Markov Chain Monte Carlo implemented Bayesian analysis was applied to estimate epistatic QTL effects. The QTL positions on the linkage maps were drawn using MapChart v2.2 software [36]. 

## Results

### Phenotypic variability and correlation

For each of the five measures of flowering time, the number of days to squaring (DS), to coloring (DC), to initial flowering (DIF), to full flowering (DFF) and to wilting (DW) from transplanting, ‘Yuhualuoying’ was later flowering than ‘Aoyunhanxiao’ in both years, and each trait showed a continuous distribution across the F_1_ population, with evidence of transgression in both directions, with the exception of DFF in 2009 (Table 1). The broad sense heritabilities of DS, DC, DIF, DFF and DW were, respectively, 0.99, 0.98, 0.96, 0.91 and 0.87 (Table 1). Each trait was significantly (P < 0.01) correlated across the two years, and each pairwise combination of traits showed a significant (P < 0.01) and positive correlation with one another (Table 2).

**Table 1:** Descriptive statistics and broad sense heritability of flowering time in chrysanthemum, based on performance of the F_1_ mapping population bred from the cross ‘Yuhualuoying’ (P1) x ‘Aoyunhanxiao’ (P2).

	Parent		F_1_ mapping population						
Trait	P1	P2	Maximum	Minimum	Average	*SD*	Skewness	Kurtosis	h2 B
Days to squaring (DS, day)									
2008	92	41	115	39	81.28	21.04	-0.31	-1.17	0.99
2009	94	43	118	42	82.38	20.34	-0.18	-1.19	
Days to coloring (DC, day)									
2008	146	76	155	65	129.46	15.31	-1.25	3.21	0.98
2009	144	77	159	59	129.23	15.15	-1.31	3.84	
Days to initial flowering (DIF, day)									
2008	155	96	167	79	139.09	13.14	-1.15	3.55	0.96
2009	157	94	168	76	142.47	13.77	-1.07	3.86	
Days to full flowering (DFF, day,)									
2008	162	102	174	87	144.13	12.12	-1.10	3.86	0.91
2009	165	99	180	106	150.64	11.09	-0.59	2.37	
Days to wilting (DW, day,)									
2008	173	108	182	103	151.82	11.66	-0.62	3.10	0.87
2009	177	107	188	105	159.64	12.56	-0.47	2.60	

*SD* standard deviation; h2 B the broad-sense heritability calculated from the mean of each flowering time trait over the two cropping years.

**Table 2:** Inter-trait Pearson correlation coefficients.

Trait	DS	DC	DIF	DFF
DC	0.67^**^			
DIF	0.70^**^	0.93^**^		
DFF	0.66^**^	0.90^**^	0.95^**^	
DW	0.67^**^	0.89^**^	0.97^**^	0.96^**^

DS days to squaring, DC days to coloring, DIF days to initial flowering, DFF days to full flowering, DW days to wilting of inflorescence, from transplanting; ** Significant correlation at *P* < 0.01.

### QTL analysis

**Figure 1:**
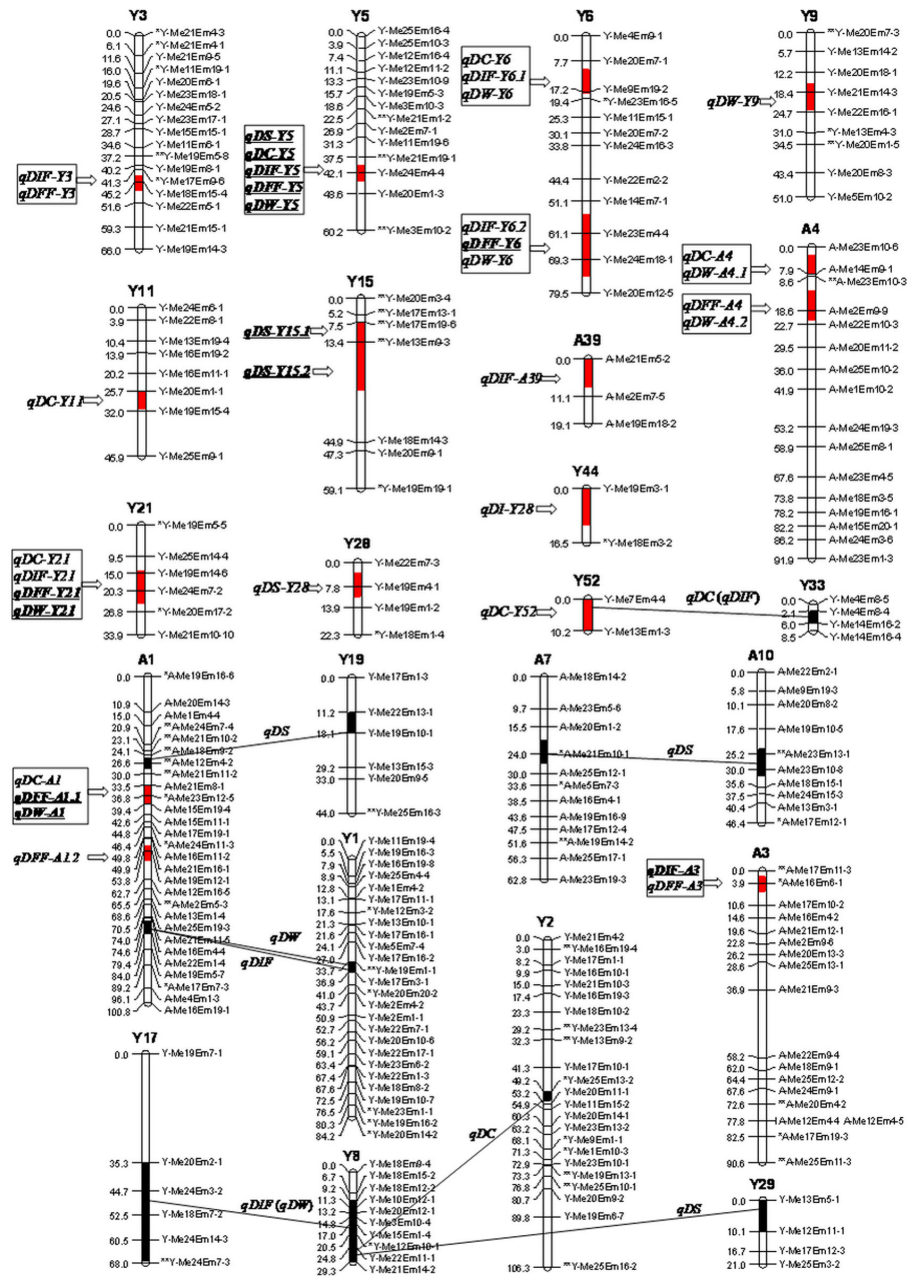
Graphical representation of additive and epistatic QTL for selected flowering time traits. Loci shown in bold and underlined represent those detected in both cropping years. Lines linking pairs of loci indicate additive x additive epistatic QTL. Genomic regions outlined in red harbor QTL with additive effects, whereas those in black harbor those which interact epistatically. Genomic regions containing QTL clusters are boxed. Days from transplanting to squaring (DS), coloring (DC), initial flowering (DIF), full flowering (DFF) and inflorescence wilting (DW). ** Significant distorted segregating at P < 0.01.

In all, 35 additive QTL were detected, of which 13 were expressed in both years (Figure 1, Table 3). The range in the proportion of the phenotypic variance explained (PVE) was from 5.8% to 22.7% (Table 3). 

**Table 3:** Additive QTL for selected flowering time traits.

Trait	QTL	LG	Marker interval	2008				2009		
				LOD	*A*	*R* ^2^		LOD	*A*	*R* ^2^
DS	*qDS-Y5*	Y5	**Y-Me21Em19-1–Y-Me20Em1-3	3.5	12.8	8.7		4.3	13.7	10.7
	*qDS-Y15.1*	Y15	**Y-Me17Em19-6–**Y-Me13Em9-3	3.8	-16.1	11.9		3.3	-13.9	9.7
	*qDS-Y15.2*	Y15	**Y-Me13Em9-3–Y-Me18Em14-3	4.3	-21.4	22.7		3.5	-18.6	18.7
	*qDS-Y28*	Y28	Y-Me22Em7-3–Y-Me19Em1-2					3.5	-12.2	8.7
DC	*qDC-Y5*	Y5	**Y-Me21Em19-1–Y-Me20Em1-3	3.1	8.6	7.6		2.7	8.0	6.6
	*qDC-Y6*	Y6	Y-Me20Em7-1–*Y-Me23Em16-5	2.8	-8.1	6.7				
	*qDC-Y11*	Y11	Y-Me20Em1-1–Y-Me19Em15-4	3.0	-9.2	9.0				
	*qDC-Y21*	Y21	Y-Me19Em14-6–*Y-Me20Em17-2					2.5	8.1	6.9
	*qDC-Y52*	Y52	Y-Me7Em4-4–Y-Me13Em1-3	3.1	-9.1	8.6				
	*qDC-A1*	A1	A-Me21Em8-1–A-Me15Em19-4	3.0	10.1	9.7				
	*qDC-A4*	A4	A-Me23Em10-6–A-Me14Em9-1					2.7	-9.32	9.4
DIF	*qDIF-Y3*	Y3	*Y-Me17Em9-6–Y-Me22Em5-1	3.2	-7.3	7.4				
	*qDIF-Y5*	Y5	**Y-Me21Em19-1–Y-Me20Em1-3	4.4	8.8	10.4		4.4	9.1	10.6
	*qDIF-Y6.1*	Y6	Y-Me20Em7-1–Y-Me9Em19-2					3.6	-9.0	10.0
	*qDIF-Y6.2*	Y6	Y-Me14Em7-1–Y-Me24Em18-1	4.0	9.1	10.7				
	*qDIF-Y21*	Y21	Y-Me19Em14-6–*Y-Me20Em17-2	3.4	7.9	8.8				
	*qDIF-Y44*	Y44	Y-Me19Em3-1–*Y-Me18Em3-2					2.7	7.6	7.38
	*qDIF-A3*	A3	**A-Me17Em11-3–A-Me17Em10-2	2.9	-7.6	7.7		2.7	-7.5	7.0
	*qDIF-A39*	A39	A-Me21Em5-2–A-Me2Em7-5	3.5	9.8	12.7				
DFF	*qDFF-Y3*	Y3	*Y-Me17Em9-6–Y-Me22Em5-1	2.5	-6.0	5.8				
	*qDFF-Y5*	Y5	**Y-Me21Em19-1–Y-Me20Em1-3	4.1	7.8	9.7		3.2	6.4	7.6
	*qDFF-Y6*	Y6	Y-Me14Em7-1–Y-Me20Em12-5	4.0	8.4	11.1		19.6	6.2	7.0
	*qDFF-Y21*	Y21	Y-Me19Em14-6–*Y-Me20Em17-2	4.4	8.5	11.5		4.9	8.8	15.1
	*qDFF-A1.1*	A1	A-Me21Em8-1–A-Me15Em19-4	3.5	8.5	11.0		4.4	8.6	13.2
	*qDFF-A1.2*	A1	A-Me16Em11-2–A-Me21Em16-1					2.8	-6.2	7.2
	*qDFF-A3*	A3	**A-Me17Em11-3–A-Me17Em10-2	2.5	-6.5	6.5				
	*qDFF-A4*	A4	**A-Me23Em10-3–A-Me22Em10-3	2.8	-6.7	7.2				
DW	*qDW-Y5*	Y5	**Y-Me21Em19-1–Y-Me20Em1-3	4.4	7.8	10.4		4.4	8.3	10.5
	*qDW-Y6.1*	Y6	Y-Me14Em7-1–Y-Me24Em18-1	4.3	8.0	10.9				
	*qDW-Y6.2*	Y6	Y-Me20Em7-1–*Y-Me23Em16-5					3.4	-7.7	8.9
	*qDW-Y9*	Y9	Y-Me20Em18-1–Y-Me22Em16-1					2.5	-7.4	7.9
	*qDW-Y21*	Y21	Y-Me19Em14-6–*Y-Me20Em17-2	5.2	9.0	14.3		3.2	7.8	8.6
	*qDW-A1*	A1	A-Me21Em8-1–A-Me15Em19-4	3.4	7.9	10.3		6.7	6.7	6.8
	*qDW-A4.1*	A4	**A-Me23Em10-3–A-Me22Em10-3	2.9	-6.6	7.7				
	*qDW-A4.2*	A4	A-Me23Em10-6–A-Me14Em9-1					2.9	-7.8	9.4

LG: linkage group, LOD: maximum log of odds score, *A*: additive effect, *R* ^2^: percentage of phenotypic variance explained by each QTL. Days from transplanting to squaring (DS), coloring (DC), initial flowering (DIF), full flowering (DFF) and inflorescence wilting (DW). * and ** indicate markers distorted at, respectively, P < 0.05 and < 0.01.

Based on the ‘Yuhualuoying’ map, four DS QTL were detected, mapping to linkage groups (LGs) Y5, Y15 and Y28. Of these, *qDS-Y5*, *qDS-Y15.1* and *qDS-Y15.2* were expressed in both years. The ‘Yuhualuoying’ *qDS-Y5* allele increased DS, while those at *qDS-Y15.1*, *qDS-Y15.2* and *qDS-Y28* decreased it. The largest effect locus was *qDS-Y15.2*, responsible for a 21.4 day delay in DS in 2008 (PVE of 22.7%) and a 22.7 day delay (PVE of 18.7%) in 2009. Five DC QTL were identified from the ‘Yuhualuoying’ map and two from the ‘Aoyunhanxiao’ map, but only one of these (*qDC-Y5*) was expressed consistently. The PVE associated with each locus was < 10%. Some of the ‘Yuhualuoying’ alleles acted to increase DC and others to decrease it (similarly for the ‘Aoyunhanxiao’ alleles). Eight DIF QTL were detected, of which *qDIF-Y5* and *qDS-A3* were expressed in both years. Some of the ‘Yuhualuoying’ alleles acted to increase DIF and others to decrease it, and similarly for the ‘Aoyunhanxiao’ alleles. The largest effect locus was *qDIF-Y5*, which delayed DIF by 9.0 days (PVE of >10%). A DFF QTL was mapped to each of LGs Y3, Y5, Y6, Y21, A3 and A4, and two on LG A1. Of these, *qDFF-Y5*, *qDFF-Y6*, *qDFF-Y21* and *qDFF-A1*.*1* were expressed in both years, with the latter two each associated with > 10% of the PVE. The ‘Yuhualuoying’ alleles increased DFF, while the ‘Aoyunhanxiao’ ones increased DFF at *qDFF-A1*.*1* but decreased it at the other three loci. The eight DW QTL detected were associated with a PVE in the range 6.8-14.3%. Some of the ‘Yuhualuoying’ alleles acted to increase DW and others to decrease it, and similarly for the ‘Aoyunhanxiao’ alleles. Loci *qDW-Y5*, *qDW-Y21* and *qDW-A1* were consistently expressed, whereas only *qDW-Y5* was associated with PVE >10% across the two years. 

### QTL clusters

**Figure 2:**
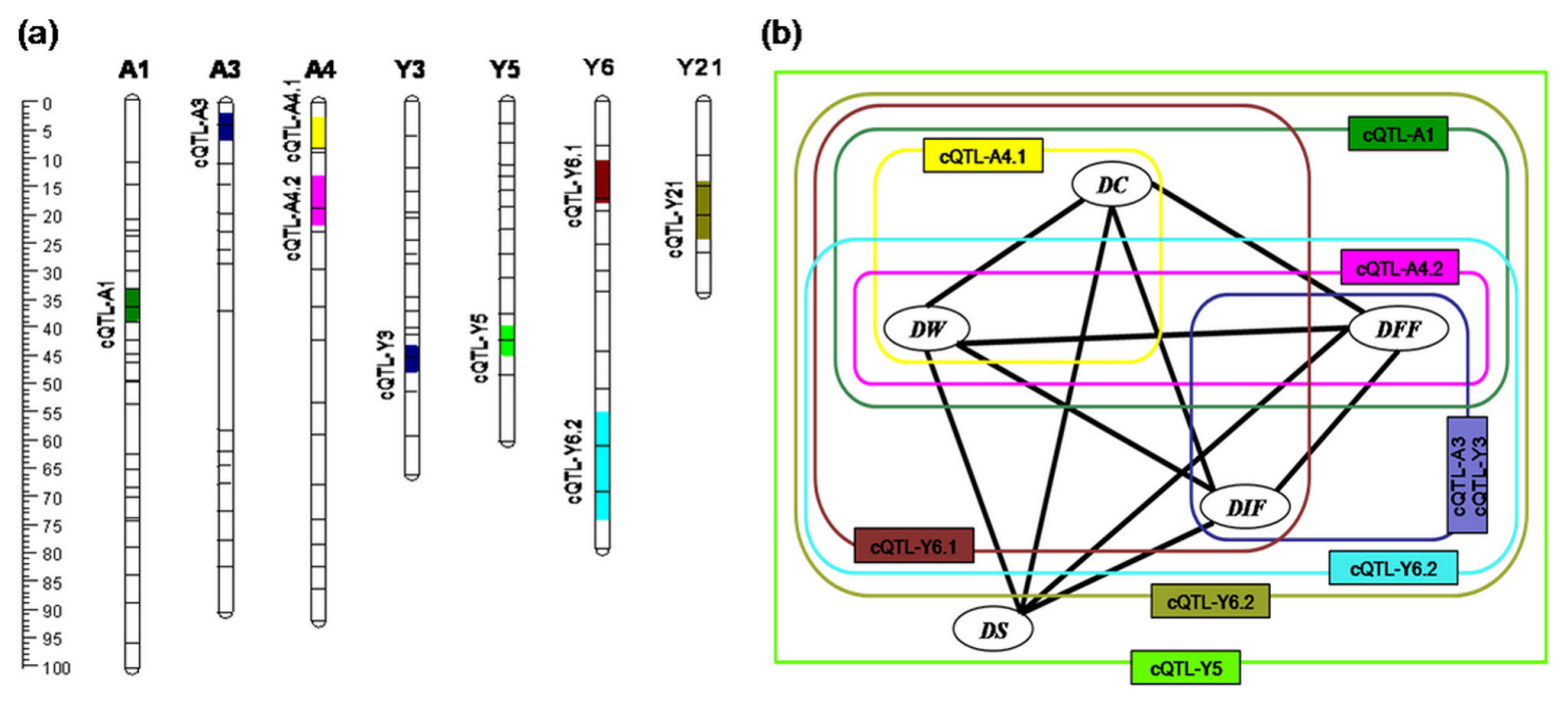
The clustered QTL and their relationship to the inter-related flowering time traits. (a) Distribution of clustered additive QTL on the linkage maps. The bar on the left shows the scale in cM. (b) Correlations between flowering time traits and the clustered QTL underlying these traits. Significantly correlated traits (P < 0.01) are connected by thick lines. Days from transplanting to squaring (DS), coloring (DC), initial flowering (DIF), full flowering (DFF) and inflorescence wilting (DW).

The nine QTL clusters (cQTL) identified mapped to LGs A1, A3, A4, Y3, Y5, Y5 and Y21 (Figures 1, 2a). The members of each cluster affected at least two of the flowering time traits (Figure 2b). The cluster on LG A1 was associated with DC, DFF and DW, and included the loci *qDIF-A1*.*1* and *qDW-A1*, both of which were expressed in both years. The cQTL on LGs A3 and Y3 included QTL for DIF and DFF, both showing decreased additive effects. Each two cQTL identified on LGs A4 and Y6 harbored additive QTL exhibiting additive effect in both directions, respectively, for different flowering time traits. The cluster on LG Y5 was associated with all five flowering time traits, and the individual loci in this genomic region were expressed in both years but showing increased additive effects. The cluster on LG Y21 was populated by QTL for four of the five traits (the exception was DS), and included the two loci *qDFF-Y21* and *qDW-Y21* which were expressed in both years. 

### Digenic epistatic QTL

The ten pairs of epistatic QTL identified are listed in Table 4. Four of the five traits were affected (the exception was DFF), and each pair was associated with a PVE of 3.5-13.9%. Three of the pairs, mapping to LGs A7-A10, Y8-Y29 and A1-Y19, affected DS. The former two delayed DS by 17.8 and 56.2 days, respectively, while the latter accelerated it by 38.5 days. The two epistatic DC QTL were located on LGs Y2-Y8 and Y33-Y52, the former accelerating DC by 42.3 days and the latter by 60.9 days. The PVE associated with the three DIF epistatic pairs, harboring on LGs Y8-Y17, Y33-Y52 and A1-Y1, varied from 5.1% to 13.9%. Of the two epistatic DW QTL mapping to LGs Y8-Y17 and A1-Y1, the former pair accelerated DW by 30.2 days and the latter by 24.8 days (Figure 1, Table 3). 

**Table 4:** Digenic epistatic QTL for selected flowering time traits.

Trait	LG	Marker interval	Position	Range	*AA*	SE	*P*	*h* 2 (aa)
DS	A7	*A-Me21Em10-1–A-Me25Em12-1	24.0	19.5–27.0	17.8	3.1	0.000	8.9
	A10	**A-Me23Em13-1–A-Me23Em10-8	27.2	23.6–32.0				
	Y8	Y-Me22Em11-1–Y-Me21Em14-2	28.8	24.8–28.8	56.2	11.3	0.000	11.3
	Y29	Y-Me13Em5-1-Y–Me12Em11-1	1.0	0.0–10.1				
	A1	**A-Me18Em9-2–**A-Me12Em4-2	26.1	25.1–28.6	-38.5	6.8	0.000	8.4
	Y19	Y-Me22Em13-1–Y-Me19Em10-1	17.2	11.2–18.1				
DC	Y2	*Y-Me25Em13-2–Y-Me20Em11-1	52.2	49.2–52.2	-42.3	11.5	0.000	3.5
	Y8	*Y-Me12Em10-1–Y-Me22Em11-1	24.5	20.5–24.8				
	Y33	Y-Me4Em8-4–Y-Me14Em16-2	3.1	2.1–6.0	-60.9	8.5	0.000	11.0
	Y52	Y-Me7Em4-4–Y-Me13Em1-3	1.0	0.0–10.0				
DIF	Y8	Y-Me15EM1-4–*Y-Me12Em10-1	18.0	17.0–20.5	-29.1	6.4	0.000	5.1
	Y17	Y-Me24Em3-2–Y-Me18Em7-2	45.7	44.7–52.5				
	Y33	Y-Me4Em8-4-Y–Me14Em16-2	5.1	2.1–6.0	-57.3	7.7	0.000	13.9
	Y52	Y-Me7Em4-4-Y–Me13Em1-3	1.0	0.0–10.0				
	A1	A-Me16Em4-4–A-Me22Em1-4	78.6	7.6–9.4	-35.5	5.5	0.000	11.4
	Y1	**Y-Me19Em1-1–Y-Me17Em3-1	36.7	33.7-36.7				
DW	Y8	Y-Me15Em1-4–*Y-Me12Em10-1	18.0	9.2–28.8	-30.2	6.17	0.000	5.6
	Y17	Y-Me24Em3-2–Y-Me18Em7-2	45.7	35.3–67.5				
	A1	A-Me16Em4-4–A-Me22Em1-4	78.6	75.6–79.4	-24.8	5.1	0.000	6.3
	Y1	**Y-Me19Em1-1–Y-Me17Em3-1	34.7	33.7–36.7				

LG: linkage group, Ranges: the position support 1-LOD intervals of each QTL, *AA*: additivity-addivity effect, *h* 2 (aa): the % phenotypic variations explained by *AA*. Days from transplanting to squaring (DS), coloring (DC), initial flowering (DIF), full flowering (DFF) and inflorescence wilting (DW). * and ** indicate markers distorted at, respectively, P < 0.05 and < 0.01.

It’s noteworthy that the epsistatic QTL located on LGs Y33-Y52, A1-Y1 and Y7-Y18 affected different flowering time traits and a genomic region could interact with several other LGs, for example, the LG Y8 locus was involved in epistatic interactions with those on LGs Y2, Y17 and Y29 (Figure 1). Additionally, unlike the other epistases involved in the background markers the epistatic QTL on LGs Y33-Y52 for DC took place between background markers and additive QTL (*qDC-Y52*). 

## Discussion

Little of the recent genetic research in chrysanthemum has focused on elucidating the basis of flowering time, but with the advent of well populated genetic maps, many quantitative traits including flowering time have begun to become amenable to genetic analysis via QTL mapping [27,32,37,38]. Flowering time, as has been shown previously by De Jong [24] and confirmed in the present research, is a highly heritable trait. Along with their high broad sense heritabilities (all above 0.85), the various flowering time traits were significantly correlated with one another, simplifying the manipulation of flowering time by breeding, whether or not a marker-assisted strategy is employed. The genetic analysis presented here, in revealing the location of a number of additive QTL controlling flowering time in chrysanthemum, at the same time has identified some potentially useful markers in the context of marker-assisted selection: for instance, SRAP markers linked to *qDS-Y15.2* which accelerated DS by about 20 days, and those linked to *qDIF-Y5*, *qDFF-Y21*, *qDFF-A1*.*1* and *qDW-Y5* which all lengthened the time taken for the plants to reach flowering.

Nine QTL clusters, each harboring loci controlling at least two of the flowering time traits, were mapped to seven of the LGs (Figure 2a); the co-location of many of these QTL is predictable given the high inter-trait correlations, which varied from 0.66 to 0.97 (Table 2). Clusters of QTL can arise through either pleiotropy or linkage [30,39-41]. By reducing a mapping interval in rice containing a QTL cluster to below 1 Mbp, Wang et al. [41] concluded that the various QTL present reflected the action of pleiotropy rather than linkage. Such a level of genetic resolution is not as yet possible in chrysanthemum due to its difficulty in fine mapping and creating advanced lines. Though, since linkage becomes more likely when genetic analysis uncovers linkage between loci controlling unrelated traits (e.g. disease resistance and flowering time) and here that the five inter-correlated traits are measuring the development of flowering time, we would suggest that the QTL clusters are much more likely the result of pleiotropy than of linkage. 

Zhang et al. [25] identified 10 and 12 SRAP markers with minor explained genotypic variations for initial blooming time (i.e. DIF in this study) and flowering duration using marker-trait analysis; however, few of them, except for the locus M20E1-1 for the two traits, were confirmed for DC rather than DIF using composite interval mapping method in this study. The very differences between the two results should be ascribed to the variant efficiency of the two methods in detecting QTL especially with minor effect. By comparison with previous researches, we also find that the LG Y5 cluster harbors not only QTL for all five flowering time traits, but also, according to Zhang et al. [25], the QTL affecting both plant height and plant width. Similarly, the LG Y21 cluster carried QTL for DC, DIF, DFF and DW herein, and the QTL underlying leaf width [26] as well. In addition, the LG Y29 harboring QTL for leaf width [26] has an interaction with the genomic region on LG Y8 that epistatically underlies DS. These findings should contribute to the significant correlation between flowering traits investigated herein and above-mentioned traits (Table S1). Also, as ‘Yuhualuoying’ includes positive alleles for all these traits, this cluster represents a potentially valuable target on marker-assisted selection for these traits simultaneously. 

Besides additivity, epistasis that refers to interactions between alleles from two or more loci in the genome underlies the genetic control of flowering time in many crops [9,10,42]. Epistatic interactions can involve pairs of additive QTL, an additive QTL with a background locus, or a pair of background loci [43,44]. Here, nine of the ten epistatic pairs involved an interaction between complete background loci, the exception involving *qDC-Y52* (an additive QTL) with a background locus. This indicates many loci that have no additive effect do influence the expression of flowering time through their interaction with other loci, and then difficult to predict the phenotype simply by the sum of the additive QTL, but rather depends on the specific combination of loci. For most traits and crops, the PVE associated with epistatic QTL tends to be much smaller than that associated with additive QTL [45]. Here, the range in PVE of the epistatic QTL was lower than that of the additive QTL (3.5-13.9% *vs* 5.8-22.7%). As was also the case for QTL determining plant architecture and leaf size [25,26], epistasis appears to be of only minor importance in chrysanthemum. Noteworthily, the identified epistatic QTL for flowering time exhibited a large effect (17.8-60.9 day), so it is not surprising that over-parent individuals predominate in the F1 population herein. In the context of chrysanthemum improvement, the additive x additive epistatic QTL, some of which were of considerable effect, are in principle selectable. 

The detection of QTL for traits of relevance to crop improvement offers the potential for so-called “knowledge-based” breeding [1]. Our concentration on the mapping population derived from the cross ‘Yuhualuoying’ x ‘Aoyunhanxiao’ reflects a major parental contrast for flowering time, plant architecture, inflorescence form and a number of other breeder's traits [25-27,32,37,38]. Flowering time is generally associated with high heritability, irrespective of the complications introduced by polyploidy. Thus defining the major QTL underlying this trait is a worthwhile undertaking. Once identified, the effect of these loci will need to be validated across genetic backgrounds and environments [42,46,47], and following this, allelic variation can be explored by association mapping in diverse germplasm [48]. The present study has confirmed the quantitative nature of flowering time in chrysanthemum. The nine QTL clusters identified, whether they arise via pleiotropy or linkage, reflect the highly correlated nature of the five flowering time traits. The major QTL, and in particular the QTL cluster on LG Y5, are currently being targeted in our ongoing efforts to streamline the improvement of chrysanthemum via the use of molecular methodology. 

## Supporting Information

Table S1 **Correlations between flowering time traits investigated in this study and other traits.** Days from transplanting to squaring (DS), coloring (DC), initial flowering (DIF), full flowering (DFF) and inflorescence wilting (DW).
(XLS)
